# Progress of the Medical Sciences

**Published:** 1891-06

**Authors:** 


### Progress of the Medical Sciences.

### MEDICINE.

The question of the value of tuberculin in the treatment of internal tuberculosis, and especially of pulmonary phthisis, still attracts a great share of medical attention, and during the last three months numerous papers have appeared. The ultimate position of the treatment still remains uncertain, but a more extended experience seems to show that it will be but of limited utility. It was to be expected that the outburst of enthusiasm with which the announcement of Koch's discovery was received would be succeeded by a period of corresponding reaction. The pendulum has swung back in the opposite direction, but we may hope that it will shortly come to rest and the real value of the remedy be satisfactorily ascertained. In the face of the well-authenticated cases, relatively few in number as they may be, in which remarkable benefit, if not complete cure, has been the direct result of the injections, it is impossible to conclude, as some authors would have us, that tuberculin is incapable of doing good and too potent for harm to make its employment desirable. The dangers of the treatment seem to have been exaggerated, as with properly selected cases and careful watching during the administration of tuberculin there appears to be little risk of accident.

On the other hand, we have learned enough of the remedy to know that it must be used with circumspection; that, although the evidence of its value in early pulmonary phthisis is too strong to be doubted, even in the early stage the results are often disappointing and occasionally bad, whilst in the advanced stages of the disease the ill effects more than counterbalance the advantages. In the accounts of cases, it is often mentioned that after injection signs of consolidation were observed in parts of the affected or of the opposite lung, in which no signs of disease had been previously detected: these signs are taken as indicative of local reaction around foci of tubercle previously too minute for recognition clinically. Dr. Heron states that in his cases these signs all cleared up before the patients left the hospital. The point is an important one, and is often not brought out with sufficient clearness in published reports, as we require to be certain that this clearing-up is the constant result before we can use tuberculin with confidence.

The Official Report of the German Government—which, however, it must be remembered, only went up to the end of 1890, and therefore only included the early experiences—was a disappointing one, after the hopes that had been raised. In this country the divergence between the opinions of different observers is very great. Some condemn the treatment altogether—Dr. Theodore Williams, for instance, thinks that it is inferior to the ordinary methods of treatment. One of the chief hindrances to success is the difficulty of obtaining constitutional tolerance of tuberculin without the loss of the local effect on the lesions. Mr. Watson Cheyne, in his very valuable and moderate statement, recommends a continuous method of administration, by which he believes this result will be best attained. He also thinks that the remedy will prove to be of greater value in medicine than in surgery. His mode of administration would seem, however, to be inconvenient to carry out in ordinary practice, especially as he considers that, to ensure a satisfactory termination in phthisis, the duration of treatment should be two years.

Under such conditions, we may perhaps ask for the demonstration of more decided gains than have yet been shown. It seems probable, however, that further experience will show that tuberculin is the most potent curative agent we possess for certain forms of phthisis, and will bring out the best method of employing it.

Meanwhile, with regard to the line of action to be taken at present; in early cases of phthisis, injections of tuberculin, combined with ordinary modes of treatment, seem to offer the best prospect of cure. Dr. Douglas Powell has pointed out that in early cases in which there are small tuberculous centres, with a catarrhal condition of the surrounding tissues, tuberculin may possibly hasten fibroid formation; but that having obtained some success, it is not wise to continue the treatment too long, but rather to trust to hygienic and general measures to complete the obsolescence of the tubercles. In cases which have passed beyond the early stage, it is more difficult to decide; but if the patient is steadily losing ground under the ordinary methods of treatment, tuberculin should be tried, after the conditions have been explained to him, as offering, at any rate, a prospect of amelioration.

Dr. Arthur Ransome[1] has carried out some experiments to test the influence of exposure to light, of fresh air, and of a dry soil upon the virulence of the tubercle bacillus. He selected two specimens of sputum—one containing few, the other many bacilli; Portions of each were exposed in three ways: (1) in a watch-glass; (2) in cotton-wool cages; (3) in a flask arrangement with cotton-wool. After exposure under the following conditions, the sputum was injected into rabbits, and its virulence determined by its power of inducing tuberculosis in these animals. None of four specimens exposed to fresh air and light on a dry soil, in each of the three arrangements above mentioned, conveyed the disease; but one of three portions exposed in darkness to otherwise similar conditions, produced tuberculosis. Of two specimens exposed in the light on the window-sill of a four-roomed tenement-cottage in Manchester, situated on a clay soil, badly ventilated and without cellarage, one produced tubercle, the other did not: an identical result was obtained in the case of two specimens exposed in the same cottage, but in comparative darkness. A specimen exposed in the ventilating shaft from a ward of the Bowden Hospital for Consumption conveyed the disease, but a portion of the same after ten days' exposure to the action of ozonised oxygen failed to do so. Although the experiments were few in number, he thinks that the conclusion may be drawn that fresh air, light, and sandy soils have a distinct influence in arresting the virulence of the bacillus; darkness interferes with this antiseptic action, but mere exposure to light in otherwise bad sanitary conditions does not destroy the virus.

From an analysis of several cases, Professor Lancereaux concludes that a pulmonary lesion may exist in the child or in the adult as a result of hereditary syphilis. This lesion, so far as is at present known, is localised in one lung, affects the lower lobes, and takes several years to evolve. The physical signs are, dulness, with (in some cases) tubular or cavernous breath-sound, and abundant expectoration. *Post mortem* a sclerosis of the pulmonary parenchyma is found, with moderate-sized cavities circumscribed by cicatricial tissue. Tracts of fibrous tissue give the lung a certain analogy with tuberculous lungs; there is, however, a complete absence of tubercles in both the sound and morbid parts of the lung. As there is no dilatation of the bronchi, the cavities must originate in the softening of gummatous tumours or in

[1] *Medical Chronicle*, March, 1891.

the partial necrosis of the sclerosed pulmonary tissue. These morbid changes are remarkable for their slow evolution, and sometimes persist for years with appreciable change in the local and general health. If taken early, they are amenable to treatment by mercury and iodide of potassium; the treatment should be carried out energetically. The pulmonary lesions differ from those of acquired syphilis only in the more complete organisation of the syphilitic growth and by its greater diffusion and extension.

The tendency of the investigations of recent years has been to attach increasing importance to lesions of the pancreas in diabetes, more especially since v. Mering and Minkowski produced diabetes in dogs by extirpation of the pancreas. They found however, that if a small portion of the gland were left, diabetes did not occur even though the excretory duct were completely removed. Lépine states[1] that healthy blood possesses what he calls glycolytic properties. Fresh blood contains a considerable proportion of sugar; if it be allowed to stand for an hour at the body temperature, 20-40 per cent. of the contained sugar disappears. He considers that this glycolytic property is due to a ferment contained in the corpuscles, more especially in the white corpuscles, since portions of blood richest in leucocytes contain most ferment, and the chyle is as active in this respect as the blood itself. In animals deprived of the pancreas and in cases of diabetes, this glycolytic power falls to 5, 2, or 1. The pancreas thus seems to be the chief source of the ferment. He supposes that the activity of the pancreatic cell manifests itself in two directions—in formation of the pancreatic juice which is poured into the excretory duct and of the glycolytic ferment, secreted by the opposite pole of the cell and passed into the blood lymph. In diabetes or in extirpation of the pancreas, the absence of this glycolytic ferment from the blood allows sugar to accumulate in it.

In his recent lectures on the "Neuroses of Development," Dr. Clouston is inclined to attach importance to the shape of the palate as an index of brain development. He classifies palates into three groups according to their shape, of which the first, the typical or "horse-shoe" arch, and the second, the neurotic or "gothic" arch, occur in about equal proportions in the healthy; the third variety, or "deformed" palate,

[1] Abstract in *Brit. Med. Journ.*, April 4, 1891, from *Revue Scientifique*.

has for its most common form a V or saddle-shape, produced by the presence of a bony shoulder alongside of the teeth on each side, but also includes palates in which there are marked central bulgings, depressions, or cup-shaped hollows. In 604 sane persons, some variety of the "deformed" palate was found in only 19 per cent.; while in 161 persons either idiots or suffering from some congenital mental defect, it was present in 61 per cent. and of cases of acquired insanity, in the insanity of adolescence, which shows the most marked heredity, in 55 per cent. The important relation of the palatal arch to the base of the skull in man is shown by the fact that a perpendicular let fall through the most anterior part of the brain passes through the middle of the palate, whereas in the monkey it only touches its posterior margin. On account of the close relationship of the upper maxilla to the base of the skull, and necessary dependence of the skull-case and palate on the development of the brain, the shape of the palate may afford an index to the latter. If brain-growth dominates skull-growth in all parts, it will determine the development of the palate; and the size and shape of the brain being governed by hereditary influences, a bad nervous heredity will declare itself both in a badly-formed brain and in an abnormal palatal arch. The deformed palate is not an expression of the reversion to a lower type of skull; for in savage races the shape of the skull, if not altered from disease or degenerative changes, does not modify the dome of the palate nor convert it into the V-shape. The shape of the palate makes no difference to the sense of smell nor to the power of mastication, but alters very materially the facial expression and slightly affects the speech.

He considers that the vaulted palate and altered dental arch are to be taken with other abnormal changes in the head, and especially in the facial expression, as morphological indications of defective development; and that the change in the shape of the palate may possibly be explained by the supposition that the frontal lobes, the part of the brain which lies above the palate, become arrested in development through bad heredity, and the base of the skull in front is therefore narrowed, while at the same time the jaw must remain large enough to hold the normal number of teeth.

Prof. Lannelongue[1] has performed craniectomy in twenty-five children, some being cases of microcephalic idiocy, and the others presenting various cerebral troubles with or with-

[1] *L'Union médicale* and *Nouv. Icon. de la Salpêtrière*, March, 1891.

out epileptic attacks. He argues that among microcephalics the ossification of the cranial sutures takes place too early—in one infant the fontanelles were ossified at birth,—and that even if it is admitted that the rate of ossification depends on cerebral growth and activity, it is not the less true that the occurrence of premature synostosis will arrest in a parallel and definite manner the growth of the cerebrum. The *modus operandi* is, the removal of osseous bands in a linear direction along the course of the sutures or over the motor area of the cortex, or the separation of bony flaps by the removal of narrow strips of bone so as to leave the flaps connected with the skull by a broad or narrow base. The indications for the operation are: (1) microcephaly, (2) deformities of the skull arising from uterine compression when there is deficiency of amniotic fluid, (3) in old infantile meningeal hemorrhages, and (4) in certain forms of hydrocephalus and other cases. The operation probably acts in (1) and (2) by allowing the brain to develop and in the last by relieving pressure.

Although Prof. Lannelongue states that the results both as regards intellect and gait are good in his cases, the remote effects of the operation must be at present considered doubtful. One objection lies in the extreme difficulty of recognising premature ossification of the sutures; many cases of microcephaly which appear to be due to this cause, turning out to have the sutures non-ossified.

J. Michell Clarke.

### SURGERY.

In the last few years a great deal has been written, and some real knowledge has accumulated, on the subject of Inflammation of the Vermiform Appendix. In the April number of *Annals of Surgery*, the whole subject has been reviewed in articles of conspicuous merit by five American surgeons; and these articles may be taken as the record of our present high-water mark of knowledge on the subject. The writers are: Mynter of Buffalo, McBurney of New York, Keen of Philadelphia, Stimson of New York, and Fowler of Brooklyn.

The vermiform appendix belongs both to the physician and the surgeon, and is alternately neglected and overrated by both. Its behaviour when diseased does not readily fit into the case-books; the signs cannot be tabulated as can cardiac bruits or groin swellings. Inflammation of the vermiform appendix is a disease full of diagnostic difficulties, practical perplexities, and too numerous fatalities.

To begin with, we must deal with the Agnostic—"the experienced practitioner who has seen many cases, but not one fatal." McBurney, whose unrivalled and most successful experience in operating for the disease gives him the title to speak out, deals effectually with this gentleman. If he reads McBurney's words he may return to his studies. In the clinique of this "experienced practitioner," cases of fæcal accumulation in the cæcum represent simple appendicitis; and when perforation takes place, it is reported as death from intestinal obstruction. This deliberate and sceptical philosopher is antagonised by the enthusiastic and credulous surgeon who would "cut down" on every painful spot in the right hypogastrium; and in the disputes between these two the poor patient often comes to grief.

The disease is real enough: its diagnosis, with proper study, is not impossible; while its treatment, in competent hands, is most successful. One of our writers (McBurney) gives a list of 24 operations performed by himself, with 23 recoveries; in not one was there an error in diagnosis. These were all comparatively early operations, where diagnosis was perhaps most difficult, but where treatment was most hopeful. In late cases, where what he calls "septic paresis of the intestines" has set in, operation in a number of cases was uniformly fatal. This is my own experience, as it is that of all the other writers.

Is it possible to systematise and classify our knowledge of the disease? I think, within limits, that we can. If such system is provisional and such classification may be modified hereafter, I am confident that benefit must result from our attempts meanwhile.

In the papers referred to, several classifications of the disease are given. These agree in the main, but not in detail; indeed, the many-sided features of the disease render a simple and natural classification almost impossible.

The classification that seems to satisfy most requirements, clinical and pathological, is that of With of Copenhagen, referred to by Wynter. Modifying With's classification a little, we believe the leading varieties may conveniently be be known as I. Plastic; II. Purulent; and III. Perforative.

I. Plastic appendicitis is characterised by the development of adhesions between the appendix and neighbouring organs, and would correspond to the variety clinically known as Recurrent Appendicitis. Such adhesions may be either a few fine threads joining the appendix, usually at some point near its tip, to some abdominal organ; or they may be of the most extensive character, being general matting together of bowel, with dense fibrous tissue in the centre of which is the appendix. In one case at operation I have found quite acute symptoms produced by adhesion of the tip of the vermiform appendix to the left ovary; in another the appendix was, with much difficulty, found embedded in a mass of surrounding adhesions. The symptoms here are chronic or recurrent, with complete or partial remissions.

II. Purulent Appendicitis is characterised by the early formation of an abscess or abscesses around a perforation in the appendix. Such an abscess is protected or localised by adhesions, which prevent the diffusion of its contents through the general cavity. Secondary and outlying abscesses are formed around the first; and if the patient survives and operation is not performed, we may find around the small appendix an enormous collection of pus contained in several pouches, the walls of which are formed by adherent bowel or bare parietes. A rare form, in which the perforation takes place between the layers of the appendicular mesentery, may burrow under the peritoneum of the pelvis and right loin. This variety, though it may follow the first, is usually subacute from the beginning, and has but little remission till the disease is at its worst, in from a fortnight to three weeks.

III. Perforative Appendicitis is intended to refer to the variety in which there is a sudden discharge of fæcal matter into the abdomen, without the formation of protective adhesions. There is collapse more or less profound from the beginning of the onset which is usually without warning; and, in spite of operation, unless when performed very early, the result is almost uniformly fatal.

Recognising these as natural varieties, we must further note that they occasionally run into each other. Thus, a plastic appendicitis may become purulent; and a purulent and protected appendicitis may suddenly become diffuse. But it is certainly the case that they most frequently remain during the greater part of their progress distinct.

This is not the place in which to discuss symptomatology, but I may refer to a few signs which seem to be noteworthy.

McBurney's "point" has been made a good deal of, and has been alternately overpraised and discredited. This symptom is elicited by pressure with the point of the finger over a small area about midway between the umbilicus and the right anterior spine of the ilium. This area overlies the base of the appendix. I believe it is a sign of real value in all cases of plastic appendicitis. In the suppurative variety there is usually palpable thickening, and an area of tenderness wider than can be covered by the finger; in the perforative variety the symptom is of little value.

Palpation gives help in proportion to the tactile capacity and experience of the examining surgeon. In my opinion it is the most valuable means of physical diagnosis; and in the great majority of cases may be almost decisive. By the rectum some thickening will be found somewhere near the sacro-iliac synchondrosis, usually in the first variety, always in the second, and but rarely in the third.

The left hand pressing downwards to the right forefinger in the rectum may be of assistance, and may even prove the presence of a cystic tumour between them. Bi-manual, or rather bi-digital, examination is, in my opinion, the most valuable single means of diagnosis in the first and second varieties.

Not a few of the worst cases are diagnosed as intestinal obstruction: these are almost uniformly of the perforating variety, or of the purulent, in which a large localised abscess has suddenly become diffuse. These are, in fact, examples of intestinal paresis. If there is a large abscess in the pelvis, a frequent discharge of mucus by the rectum is sometimes met with. Very rarely is there diarrhœa.

One other misleading sign may be mentioned: this is the presence of a tympanic note over an area which palpation tells us contains a collection of pus. With this sign there may be actual or comparative dulness in the dependent portions on the left flank. In two recent operations I have found this combination of resonance on the right side, and dulness on the left; and in both cases it helped to mislead those in charge of the patients. The dulness on the left side was from fluid-laden bowels; the resonance on the right was from the presence of free gas overlying the putrid collection of pus. Here the value of palpation is conspicuous.

As to the best time for operating, opinions differ somewhat. A limit of three days has been set to the time we may safely wait. As an average over all cases taken indiscriminately, this would probably be fairly accurate; but in some cases the limit would properly be extended, and in others it would certainly be too long. Thus, in simple plastic appendicitis, with recurrent attacks, we may wait for weeks or even for months before determining to operate; while in the perforating variety operation cannot be performed too soon after the condition has been diagnosed. In the suppurating variety, which is probably the most common, waiting for two or three days may be advisable, but then only if diagnosis is uncertain. If there is a strong presumption that pus, however small in amount, has formed, operation should be done without delay. Of course if the abscess is large and palpable, the indication to operate is urgent.

The characters of the disease being so varied, the operative details must present a corresponding variety. But there is a very general harmony of opinion as to the main indications to be observed in all cases. Thus, the parietal incision should be made over the seat of disease, and not in the middle line, unless the operation is purely an exploratory one. Adhesions should never be disturbed if it is possible to avoid doing so, and irrigation should be conducted with great gentleness. Secondary outlying abscesses should, if possible, be opened and drained through the primary abscess surrounding the origin of the disease. It is wiser to be content with simple evacuation and drainage, than to run any risk of causing diffusion of pus through the cavity by elaborate attempts at thorough cleansing and disturbing of conservative adhesions.

In dealing with the appendix itself, our method of procedure will vary according as it is embedded in a mass of dense fibrous adhesions or lying free in an abscess cavity. In the worst cases it may be left to its fate, while we wait for the issue which will follow simple evacuation of an abscess: in such cases the operation will probably have been performed without an anæsthetic. In the cases of plastic appendicitis, where there are abundant adhesions, the isolation of the organ may be a very difficult and tedious business; where the adhesions are not abundant, the operation may be very simple. The easiest cases are usually those in which a small abscess surrounds the appendix. Removal is probably the best treatment in all cases. And almost any variety of closure from simple ligation with catgut to involution of the mucosa and elaborate suturning of the serosa has been proved to be successful. One point should be noted; and that is, whether there is a free passage into the cæcum. The merest drop of pus left between the ligature and the cæcal aperture may, if this aperture is not pervious, lead to a catastrophe. If there is any doubt, the appendix should be completely involuted, and the serosa of the cæcum sutured over it.

J. Greig Smith.

### OBSTETRICS.

Notwithstanding all that has been said about the duration of pregnancy, the length of a normal gestation does not seem to be definitely settled. The question is one that has a more important practical interest than is often supposed. Not only is it desirable that we should be able to give with some amount of accuracy the probable date of delivery, but in cases where the induction of premature labour is indicated, it is at times a matter of very great importance that we should be in a position to say how far the pregnancy has advanced.

Dr. James Oliver[1] thinks that an investigation regarding the human female may be greatly aided by a review of the conditions in reference to the duration of pregnancy in the lower animals; although in these, notwithstanding that the date of insemination can be fixed with certainty, the period of gestation is found to vary considerably. Doubting the assertion that the duration of normal human gestation may range from 260 to 304 days, and dissenting from the usual mode of calculating 278 days from the cessation of the last menstruation, Dr. Oliver, who quotes many observations made on the lower animals, says the best results will be obtained by first ascertaining the usual number of days in the inter-menstrual period; half this number is then to be added to the date when the last menses disappeared, and a further addition of 260 days will most probably give the date of confinement. This plan, ingeniously deduced by Dr. Oliver, is however beset with difficulties, of which the varying inter-menstrual period in the same individual is not the greatest. Any theory based on the cessation of the menses must be often fallacious. A physical sign that is tolerably constant is needed as a guide in the determination of the stage of the pregnancy.

Dr. A. P. Clarke,[2] proceeding on the theory that parturition is not a mechanical but a physiological function, considers that anything which tends to exhaust the general system interferes with the normal action of the parts, and disturbs the proper functions of the uterine tissue; and he considers that not only is uterine inertia induced by keeping the patient for a long time in one position, but that other troubles, such as cystitis with inflammation proceeding up the ureter and involving the kidney, may be a consequence. He quotes the *post mortem* record of a case in which this seemed to be the order of events. Dr. Clarke would also attribute some cases where rupture into bladder or rectum has taken place to long retention in one position. He also thinks that the

[1] *Liverpool Med.-Chir. Journ.*, Jan., 1891.

[2] *Journ. Am. Med. Assoc.*, Mar. 28, 1891, p. 334.

same factor is responsible for some of the cases in which inflammation of the uterine appendages comes on soon after delivery. Although Dr. Clarke probably overrates the influence of position, it is possible that this may be occasionally an overlooked cause of some *post partum* troubles; and it would be well to bear in mind that a frequent change of position is desirable, and may even result in bringing a labour to a speedy termination. In the discussion which followed Dr. Clarke's suggestive paper, emphasis was laid upon the advantages of chloral in tedious labour. There can be no doubt that, given in two doses of 15 grains each, twenty minutes apart, as mentioned by Dr. Playfair,[1] a first stage may be often quickly changed into a second.

In the *Journal de Médecine*[2] for February 25th, 1891, is given a detailed account of the antiseptic precautions observed in various French maternities. These are most elaborate, and the good results obtained are attributed to them. But it is clear that accoucheurs have something to learn from the recent history of operative surgery, and are more likely indebted to a strict regard for cleanliness than to the antiseptics they adopt. Although impossible in private practice, no doubt many of these precautions are excellent, and amongst them, for general adoption, may be specially recommended a lubricant consisting of perchloride of mercury and vaseline. I have for some time used such a preparation (gr. i. ad ℥ i.) and have every reason to be well satisfied with it. The baby is not without a share of these attentions, and at one of the hospitals receives, probably with disapproval, a prophylactic instillation of lemon-juice into the eyes. In all the institutions there is a routine practice of bathing the eyes with some disinfectant.

With a desire to secure complete asepticism, it has been proposed that the blade and handle of the forceps shall always be made in one piece, so that there may be no crevices in which noxious material may lurk.

On February 26th, Dr. P. McCahey exhibited before the New York Academy of Medicine[3] an instrument which he calls "The Atmospheric Tractor," and which he suggests as an occasional substitute for the forceps. It is a kind of cup, with handle made of thick, soft rubber. The

[1] *Brit. Med. Journ.*, vol. ii., 1890, p. 715.

[2] See *Medical News*, April 25, 1891, p. 470. [3] *Medical Record*, Mar. 28, p. 385.

tractor covers an area of about five square inches, and exerts a force, when applied to a wet surface like the head of the newly born, of from twenty-five to thirty-two pounds, or over one-third of the atmospheric pressure, which is fifteen pounds to the square inch. He claimed that the instrument could be applied with facility, furnished a sufficient amount of force to extract the child, was positively harmless, and could be used with perfect safety in both normal and abnormal labours. In normal labours it was employed to shorten suffering. Of course the os must be dilated to a size as great or greater than that of the tractor. Dr. McCahey had used the instrument in about twelve cases, and had delivered in from five to twenty minutes, where nature would probably have required two hours or more.

Apart from the injury which is said to be sometimes inflicted upon the child, the obstetrician may well rest satisfied with the tractive power he already possesses, and, having Simpson's long forceps, need not seek to find any substitute for that simple instrument, which can do no harm in any careful hands.

The recent literature on Puerperal Convulsions is considerable. A noteworthy contribution to it was that in our March number by Dr. Swayne, recording a series of thirty-six cases, and extolling the advantages of venesection. Not much is to be learned from the record of isolated cases, especially when no discrimination is made of the varieties upon the importance of which Dr. Swayne so strongly insists.

In a philosophic survey of the subject, Dr. James Tyson[1] points out the importance of a prophylaxis of this complication, and laments the frequency with which pregnant women are allowed to go to term without examination of the urine. He considers that Rosenstein did not overstate the condition when he said that convulsions occur in about one-fourth of all the cases of nephritis in pregnancy, and that about 30 per cent. of the eclamptic cases die. When Bright's disease is associated with pregnancy, Dr. Tyson recommends the induction of premature labour: (1) in those cases where a previous confinement was accompanied by grave accidents which may be predicted to recur, especially those in which brain-symptoms had appeared; (2) in every primipara in whom Bright's disease existed before marriage. In those cases where it is concluded to attempt to prolong gestation to the viable period or to the end of pregnancy, Dr. Tyson insists

[1] *Medical Record*, Jan. 3, 1891, p. 1.

on the freest elimination being kept up, and this through kidneys, bowels, and skin. If, in spite of all this, convulsions occur at the time of labour, he insists that, while the necessary emptying of the uterus is being carried out, blood-letting, chloroform, or chloral should be employed to diminish the danger of the convulsions. Dr. Tyson is very emphatic in saying that blood-letting is needed almost without exception in every case of uræmic puerperal convulsions, and that many a case is lost through its omission. As soon as it has been carried out as far as seems safe or judicious, chloroform or chloral should be administered to control the convulsions, or one or the other may be used simultaneously with the bleeding. Under no circumstances is the action of chloral more happy, and the rectum affords a medium of administration as prompt and efficient in its operation as the mouth, which is generally unavailable for the purpose. No less than a drachm in solution should be given at such a time by enema, and it is unwise to temporise with smaller doses.

Another view of the subject is presented by Dr. W. S. Gardiner,[1] under whose supervision there was an examination of the urine of 180 pregnant and parturient women, taken consecutively at the Maternité Hospital, Baltimore. Four of these patients had convulsions, and three of them died. Details are given of the urine analysis and of the cases of convulsions. Dr. Gardiner admits that the number of cases presented is too small for giving a dogmatic opinion, but says that if these are to be considered average cases, the following conclusions may be drawn: (1) The presence of albumen in the urine of a pregnant woman is no sufficient cause upon which to base a prognosis of probable eclampsia. (2) The failure to find albumen in the urine of a pregnant woman is no evidence of the absence, or, at least, of the continuance of the absence, of the condition that gives rise to puerperal convulsions. (3) Albumen is so frequently found in considerable quantities in the urine of patients, immediately after the appearance of puerperal convulsions, that we are justified in making the statement that the convulsions are the probable cause of the albuminuria.

Before the Obstetrical Society of London, on November 5th, 1890, Dr. Herman[2] cited four cases of pregnancy with Bright's disease without eclampsia. He pointed out that to understand the relation between renal disease in pregnant women and eclampsia of pregnancy it was necessary to com-

[1] *Therapeutic Gazette*, Jan. 15, 1891, p. 16.

[2] *Brit. Med. Journ.*, vol. ii., 1890, p. 1124.

pare cases of renal disease without eclampsia, and said that there was very little evidence either for or against the view which many held, that puerperal eclampsia was nothing else than uræmic convulsions, occurring as the result of kidney disease in pregnancy and childbed.

It is obvious that the study of this distressing and dangerous condition has not advanced very far, and that to its consideration must be brought minds capable in no ordinary degree of making correct deductions from observed facts, and of weighing evidence in the most impartial manner.

The treatment of abortion is so frequently a difficulty, and causes so much anxiety in the mind of the obstetrician, who wants to do neither too little nor too much, that anything which can elucidate the difficulty should be welcomed.

Dr. H. C. Crowell[1] referring to cases of expulsion not later than the third or fourth month, thinks that although the uterine contents may be expelled under expectant treatment, in which he would include the administration of ergot and the clearing away of clots, &c., by the finger, yet a condition of ill health often lingers afterwards, and that even the larger part of all cases of tubal troubles date from a mismanaged abortion. He quotes Winter of Berlin, who shares his views, and then sums up as follows : (1) An abortion is a pathological process, involving the premature expulsion of the fœtus and membranes from the uterine cavity, which, normally, have an existence of nine months before they shall have completed their physiological intention. (2) That such expulsion is generally incomplete when left to nature, thus exposing the patient to subsequent pathological conditions or possibly death. (3) That every case should receive a careful examination by the use of the blunt curette, in preference to the finger, as it is safe, easier of introduction, and more effective. (4) Complete removal of all membranes, maternal and fœtal, offers the greatest protection and safety to the patient. (5) Perfect asepsis and drainage is a necessary supplement to the curette. (6) Ergot has little or no effect in the treatment of cases of abortion. If used at all, it should be in the later stages to assist involution.

Many practitioners had previous to this adopted the treatment by curette, and it is evidently growing in favour. Dr. Whitehead, a medical man in New Zealand, recommends[2]

[1] *Medical News*, April 25, 1891, p. 460.

[2] *Lancet*, Feb. 28, 1891, p. 494 ; *Brit. Med. Journ.*, Mar. 21, 1891, p. 651.

for the purpose a modification of a Sims' curette, and gives a description and illustration of the instrument, which he considers admirably suited for such cases.

In the *American Journal of the Medical Sciences*[1] Dr. W. T. Lusk has a paper dealing in detail with "Life-saving Methods in Still-births," which deserves careful study. Many cases have doubtless been given over in the belief that it was hopeless to attempt to restore life, and in which perseverance might have resulted in establishing respiration.

Dr. Lusk refers to this, and opportunely reminds us that the object of medical practice is to save life, and says that he regards the rescuing of a new-born infant from impending death to be as distinctly a professional triumph as the saving of life by ovariotomy, by Cæsarean section, or by the operation for appendicitis. But he points out that in these cases of deep asphyxia no miracles are to be wrought without patience, watchfulness, and a hopeful spirit. He reminds us that in many cases the child has been temporarily restored to life, only to die of atelectasis after a few days. To guard against this fatal sequence—due in part to imperfect expansion of the lungs, in part to lobular congestion—there is no method that rivals the one of Schultze. This, which consists in attempts to produce alternate expansion and compression of the lungs by suspension and swinging of the infant, has been most recently described and illustrated in the *System of Gynæcology and Obstetrics by American Authors*—"Obstetrics," Vol. I., p. 576.

Amongst the recently reported obstetric curiosities and rarities may be quoted the following:

(1) A primigravida, six months pregnant, shot herself, the ball entering four inches to the right, and in a line with the umbilicus. When first seen she was suffering but very little from shock. She was anæsthetised with chloroform, and the wound was found to be a penetrating one. She was sitting in a low rocking-chair when she attempted suicide, the pistol being held close to the body. A probe could be passed between the muscles to Poupart's ligament. Upon laparotomy, the abdomen was found filled with blood and clots, and a ball-wound was found to the right side of the fundus of the uterus near the attachment of the Fallopian tube. This wound was closed by sutures, no wound of exit for the ball being found.

[1] Feb., 1891, p. 109.

About twenty-four hours after the injury was received the patient aborted with a six-months' fœtus. The fœtus had a ball-wound just behind the acromion process of the scapula, another above the umbilicus, and the right leg was shattered below the knee. The patient recovered in four weeks from the time of the injury.[1]

(2) Dr. Herman[2] performed Cæsarean section in a patient at full term, whose age was 37. On vaginal examination, the pelvis was found so filled with a hard elastic swelling bulging from its left wall, that the fingers could not be inserted between the right pelvic wall and the greatest diameter of the tumour. This proved to be an osteo-sarcomatous growth, the interior of which was broken down into a cavity containing blood and pus. The mother died three days after the operation, but the precise cause of death was not ascertained. The child was living.

(3) A woman in her third labour expelled the head and arms of her child, but no further advance could be made. The child's abdomen was found to be enormously distended, and when punctured was only slightly reduced in size. The child was then partially eviscerated. Both kidneys were found to be considerably enlarged and were the seat of cystic degeneration. Dr. M. H. Morrell,[3] who reports this case, says that although several cases have been recorded in which the kidneys were sufficiently large to impede labour, he could only find the account of one case in which mutilation of the child was necessary.

(4) Dr. Kingsbury[4] relates a case in which he hypnotised a young girl under 15 years of age who was in labour, and who was delivered of a child weighing 8½ lbs., being quite positive she had had no pain. She had been hypnotised once a week for three months before her confinement; and Dr. Kingsbury advises everyone who wishes to try this method to have at least six preliminary sittings, so as to accustom his patient to the process, and also to thoroughly test to what degree of hypnosis he can bring her.

Cæsarean section, with its various modifications, and the treatment of extra-uterine pregnancy have properly passed almost entirely into the hands of the abdominal surgeon.

[1] See abstract from *Southern Medical Record*, No. 2, 1891, given in *American Journal of Medical Sciences*, April, 1891, p. 430.

[2] *Lancet*, May 2, 1891, p. 986. [3] *Medical News*, Jan. 10, 1891, p. 40.

[4] *Brit. Med. Journ.*, Feb. 28, 1891, p. 460.

Current literature records many of these cases. The *Index Medicus* will furnish the references.

It seems late in the day for those entrusted with the control of Medical Education to be trying to make up their minds about the training in obstetrics required for students, and one reads with pain and dismay the record[1] of the way in which the members of the General Council of Medical Education and Registration of the United Kingdom attempted, on May 28th, 1891, to solve the difficulties connected therewith. Dissatisfied with the present training-standard in midwifery, a member brought forward a proposition in reference to the attendance of students. The discussion which followed is not likely to give us an enhanced opinion of the dignity of the General Council of Medical Education and Registration of the United Kingdom. One speaker stated that he had a feeling akin to shame in reference to existing regulations, which were described by another member as wofully inadequate. A third speaker described the result of the previous discussion on the subject as a miserable fiasco, which made him feel ashamed of the Council. A protest against this language from another member, and a further statement by another that the present standard was admirable, did not deter yet another member from characterising their own proceedings as the subject of scoff and satire; whilst another considered that the proposal then before them, requiring students to attend a lying-in institution, was absurd, apart from being impertinent. Little wonder that, with a discussion conducted on lines such as these, the members of the General Council of Medical Education and Registration of the United Kingdom did not arrive at a very intelligent conclusion. An amendment that students should attend for six months the practice of a lying-in hospital or maternity institution was carried by 18 to 7. On being put as a substantive motion, an amendment that, with certain safeguards, part attendance on twelve labours, either at an institution or under the supervision of a medical man, should be sufficient, was carried by 14 to 11. But this failed to establish itself as a definite resolution; for an amendment to adhere to the resolution of 1888 was approved by 17 votes to 8, and was afterwards formally adopted as a substantive motion. This unedifying spectacle was thus terminated, and the *status quo ante bellum* was maintained.

Attendance at labours practically unsupervised is emi-

[1] *Brit. Med. Journ.*, May 30, 1891, pp. 1184-6.

nently unsatisfactory. Preceded by a thorough teaching of theory and accompanied by a full knowledge of a trustworthy text-book, attendance at a lying-in hospital should be made compulsory; and if this were at an institution where the number of cases was fairly large, the attendance of a month, carried out under the direction of competent resident supervision, would be ample to equip the student with the confidence and knowledge necessary for facing the variations of midwifery practice with which he would be afterwards confronted.

L. M. GRIFFITHS.

### LARYNGOLOGY AND RHINOLOGY.

During the past few months there has been issued an immense amount of literature bearing on this branch of medicine and surgery. The German journals especially have published numbers of articles dealing with the treatment of tuberculosis and lupus of the upper respiratory tract, due doubtless to the fact that the International Congress was held at Berlin, and that Koch succeeded in inducing large numbers of his medical compatriots to try tuberculin (Kochii), which was given to them for purposes of experiment long before it reached the medical world at large. The treatment of tuberculosis was admirably discussed both at the Birmingham Meeting of the British Medical Association and at the International Medical Congress.[1]

Mr. Symonds concluded an admirable paper thus: (1) Tubercular disease of the larynx should be treated on the same lines as elsewhere; viz., by cauterisation or curetting. (2) That lactic acid is the best caustic. (3) That the disease can be treated by endo-laryngeal methods in most cases.

Our objects in operating are: (1) To relieve cough and dysphagia. (2) To diminish the liability to pulmonary affection. (3) To increase the rapidity of cure in those cases disposed to it, as is done by general surgeons in tubercular disease of joints.

The most suitable cases are: (1) Those with no evidence of pulmonary disease. (2) Those with severe cough and dysphagia, but with pulmonary disease that is not rapidly advancing. (3) Those with early or chronic pulmonary disease.

Several specialists who were present at the reading of Symonds's paper expressed their approval of the lactic acid

[1] *Brit. Med. Journ.*, Sept. 13, 1890.

treatment, but it was reserved for Heryng,[1] who must be regarded as the pioneer of this crusade against tubercle, to read at the Berlin Congress a masterly account of his immense experience of these cases and their treatment. He is greatly in favour of curettement of the diseased structures, and of the application of lactic acid to them.

The indications for curettement are: (1) In circumscribed infiltration of the posterior laryngeal wall and recent unilateral affection of the epiglottis. (2) In deep ulceration of the ventricular bands. (3) In sprouting ulcers of the surface or edges of the vocal cords. (4) Where the process is spreading very quickly, with much dysphagia, due to inflammatory swelling or ulceration of the epiglottis or of the posterior wall of the larynx, with no hope of cure.

He showed a good case of cicatrisation of tubercular larynx after curettement four years ago, and pathological specimens. Altogether his work is most convincing, and shows that resolute surgical procedure by scraping and lactic acid—just the treatment, in fact, that surgeons know will cure tubercular disease in other parts, *e.g.*, joints and lupus of skin and mucous membranes, will also cure the larynx.

But following rapidly on all this good work came Prof. Koch's discovery; and whilst the time is not yet ripe for attempting to fully estimate the value of tuberculin (Kochii), a great deal has been written by men competent to judge, and who have been treating patients affected with throat and nose disease for the past six months.

At the close of the important discussion on the action of tuberculin by members of the Berliner Med. Gesellschaft, Prof. B. Fränkel said that, though fully alive to the possible danger of the injections, he had found the curative effect in many cases so much better than he had yet observed to follow any other method, that, in his opinion, the physician, after having carefully and conscientiously selected suitable cases, must calmly face the danger. Fränkel is a careful observer, and his experience in February, 1891, was just that of December, 1890, when he told me that laryngeal tuberculosis could be cured by injections.

Dr. Michael[2] reports favourably of the action of tuberculin in a number of his own cases of tubercular and lupous throat mischief, and in those of other specialists, who state that ulcers clean, necrosis of previously infiltrated parts occurs, and is followed by removal of the slough and healing of the ulcer

[1] *Berl. klin. Woch.*, Sept. 15 and 22, 1890.

[2] *Journal of Laryngology and Rhinology*, Jan. and April, 1891.

left, and subsidence of infiltration takes place; and at the same time functional improvement of the voice, &c., goes on *pari passu*. This I have seen in some cases, the sloughing and after-healing being most remarkable. At the same time, some cases show signs of considerable disturbance: the voice, which previously had been full, is extinguished, because of fresh inflammatory mischief, with swelling, infiltration, and necrosis, and only after a considerable period has elapsed do the affected structures return to the condition in which they were before the treatment was begun, or even stop short of that and are left in a worse state temporarily and perhaps permanently.

Mackenzie [1] reports on seven cases of affection of lungs and larynx treated by injections; with the result that one was much improved, one improved, one not improved, and four were unfavourably affected. In three of these seven cases acute circumscribed œdema took place, but in no case was any dyspnœa produced.

Lennox Browne [2] has had 14 cases under his care, and he says: "I regret to say that up to the present I have not seen any of those visible and marked evidences of improvement in the larynx such as I witnessed at Berlin . . . but symptomatic relief has been almost constant, and in some cases quite remarkable in the rapidity of its occurrence, in its extent, and in its permanency, even where physical changes have been unfavourable." As regards the therapeutic value of the lymph, I could quote the opinions of numbers of the leading laryngologists and rhinologists of this and other countries who are still using it in carefully-selected cases of tuberculosis and lupus of throat and nose, and who keep their minds open on the subject, the undue optimism of the end of the year 1890 not having given place to the pessimism that is so prevalent in some quarters to-day. As regards its diagnostic value, the balance of evidence is in favour of its power to differentiate tubercle and lupus from other diseases which, as far as appearances go, are differentiated from them with the greatest difficulty. At the same time, I have seen tuberculin cause local redness and swelling and general reaction in a case which all observation would lead us to believe was one of tertiary syphilis of the nasal septum, and the history was also in favour of that diagnosis; but then we may have a great deal to learn as to the differences between syphilis and tubercle or lupus. I consider that the

[1] *Journal of Laryngology and Rhinology*, April, 1891.

[2] *Journal of Laryngology and Rhinology*, May, 1891.

only course for laryngologists to pursue is, to keep an open mind on the question of the value of tuberculin. At the same time, I cannot but acknowledge that the results up to the present have been disappointing; but it is quite premature to absolutely condemn it, as is the fashion with some men who have seen very little of its use in their own practice or that of others.

Prof. Oscar Liebreich has introduced cantharidinate of potash, in doses of one to two decimilligrammes injected subcutaneously, for the treatment of tuberculosis. The theory of its action is, that it causes transudation of blood-serum at the affected spot, and thus disinfects *in loco* and without producing fever or any inflammatory action; but there is the unfortunate drawback that, even in minimum doses, it causes intense vesical tenesmus and in some cases albuminuria. The remedy has not been under trial long enough to say anything as to its value.

Loeffler[1] finds that solution of corrosive sublimate, 1 in 10,000, will kill the bacilli of diphtheria, and 1 in 1,000 the cultures. Permanganate of potash 1 in 100, and chlorate of potash 1 in 20, were without effect. Chloroform water—which, as we know, will keep hypodermic solutions free from fungi growth for long, if not indefinite, periods—is very effective. He therefore recommends, as local measures during an attack, a gargle of a solution of 1 in 1,000 corrosive sublimate, or 3 per cent. carbolic acid; and inhalations of turpentine and the oils of eucalyptus, citron, &c., are useful. As a prophylactic, use a gargle of aq. chloroformi, or a solution of corrosive sublimate, 1 in 10,000.

The usual crop of papers bearing on nasal reflexes has appeared, and the question of the etiology of asthma has been not a little discussed. West[2] considers the discovery of the association of nasal affections with asthma the greatest advance in our knowledge of the pathology of therapeutics for many years past. Thorowgood has seen marked improvement after removal of nasal polypi. Hall has noticed great improvement, but never complete cure, after treating the

[1] *Deutsch. med. Woch.*, 1891, No. 10.

[2] *Brit. Med. Journ.*, Nov. 15, 1890.

nose. Semon believes that we cannot distinguish between cases where treatment directed to the nose will be beneficial or otherwise. How all this criticises Bosworth's statement that every case of asthma has some nasal trouble which is the *fons et origo mali!* At the same time, every asthmatic patient ought to have the nose examined carefully, and my experience is that some distinct nasal mischief will, in a large number of cases, be discovered.

Sedziak,[1] writing elaborately on the symptoms set up by deviations of the nasal septum and the treatment necessary for their rectification, thus summarises: (1) In fresh cases, *e.g.* result of traumatism in children, tampons of wool. (2) In somewhat older ones, where the cartilaginous septum is affected, orthopædic treatment by means of Jurasz's modification of Adams's forceps, the nasal cavity being carefully dusted with iodol during the first few days, and ivory plugs inserted to keep the septum straight. Spines and spurs he treats by means of the galvano-cautery, Bosworth's saw, or the chisel.

*On dit* that there is to be a crusade against excessive nasal surgery at the forthcoming meeting of the British Medical Association at Bournemouth. There is, I believe, too much tinkering and meddling with the nose done by some rhinologists, of whom one well-known specialist may be looked upon as the chief offender; but no one who has vainly endeavoured by insufflation, douches, &c., to reduce the abnormal sensitiveness of an inferior turbinate, and who then has recourse to galvano-cautery and completely cures the condition, doubts the efficacy of strong measures; and the removal of spurs is frequently the only remedy for obstinate nasal and naso-pharyngeal catarrh.

Stokes read a paper before the Laryngological Society, in March, 1891, on intra-laryngeal removal of an epitheliomatous growth of the larynx, which nine months afterwards had not recurred. The operations were done with the snare, and the base cauterised. This, as we know, is not the only case on record of complete removal of a malignant growth by the mouth, but it is improbable that such success can be often repeated.

Pyoktanin continues to gain favour as a remedy for some ulcerative throat and nose troubles. The list of new and

[1] *Journal of Laryngology and Rhinology*, March and April, 1891.

marvellous drugs grows longer, but we will wait until most of them—such as goat's blood and dog's serum—have had their short-lived day, and report only on those that survive the blighting influences of use and observation of effects produced.

England as readily offers a home to useless and harmful drugs with long names as she does to Polish Jews and foreign paupers.

BARCLAY J. BARON.

### OTOLOGY.

On May 10th, 1890, Dr. Menière of Paris laid his conclusions concerning aural complications of *La Grippe* before the French Otological and Laryngological Society. He states that these complications are the result of retro-nasal affections. Of 57 cases, 23 lasted four or five weeks, 16 lasted three months, 8 lasted four months, and others were still under treatment by reason of complications, such as periostitis and mastoid inflammation. The treatment consisted in warm-water irrigation in the external canal and in the Eustachian tube, paracentesis of the drum membrane in some cases, and in four instances thermo-cauterisation of the mastoid.

Sir Wm. Dalby sounds a timely note of warning in reference to "Bubble" Remedies in Aural Surgery. Under this heading he includes: (1) The promiscuous use of artificial ear-drums, for the manufacture and sale of which a company was recently floated in London with a capital of £100,000. (2) The use of electricity by all sorts of incompetent persons. (3) The mutilation, by removal, of the turbinated bones of the nose for deafness, in cases where the nose is perfectly healthy and unaffected. (4) The hypodermic injection of pilocarpine in totally unsuitable cases. (5) The division of the tensor tympani muscle for deafness. This last he describes as including in its history "a flight of the imagination, a brief notoriety, and a burial in oblivion so rapid as falls to the lot of few achievements in surgery."

Politzer lays down the following rules in regard to the administration of pilocarpine injections for deafness: (1) The subcutaneous injection of pilocarpine is particularly indicated in recent affections of the labyrinth, be they syphilitic or not. (2) The injections are of little use in acute inflammation of the middle ear. (3) They are decidedly contra-indicated in cases of dry sclerotic catarrh of the middle ear. (4) Injections of several drops of a two per cent. solution into the tympanic cavity through a catheter are beneficial in some cases of catarrh with swelling and slight secretion.

In such cases the injections should be given for from one to two weeks alternately with Politzer's method of inflation.

Dr. Syenes, an otologist of Buda-Pesth, has found menthol of value in the treatment of furuncles of the auditory canal. A 20 per cent. solution of it is used, and a pledget of cotton wet with it is placed in the canal.

The following has been recommended by Kirchner for the same affection: Corrosive sublimate, three-quarters of a grain; water, one fluid ounce; glycerine, five fluid drachms.

Syenes found in the last epidemic of influenza that in the first stage of acute inflammation of the tympanum a 15 per cent. solution of carbolic acid in glycerine did good service.

Creolin and iodol are unsatisfactory in ear disease; salicylate of bismuth and aristol are of as little service; lactic acid, even in weak solutions, causes pain and erosions, so it soon has to be given up; cocaine is useless for tinnitus.

As an instance of the occasional danger of syringing the ear, Dr. Philips of Savannah reports a case of a man, 54 years old, with an accumulation of cerumen in the left meatus. With a small rubber-ball syringe, holding about 1½ ounces, Dr. Philips began to syringe the canal. Several syringefuls, forced gently into the ear, not bringing away the wax, he used a little more pressure. Suddenly something seemed to give way; water poured from the left nostril, and the patient complained of some pain in the ear. The wax, which was loosened, having been removed, a recent rupture of the drum membrane became visible, in the form of a thin crack, with a few drops of blood scattered along it. The wound rapidly healed under suitable treatment, and no bad after-effects resulted.

W. H. Harsant.

